# The risk of translaminar screw fixation to the transverse foramen of the lower cervical spine: a computed tomography study

**DOI:** 10.1038/srep46611

**Published:** 2017-04-21

**Authors:** Ganggang Kong, Wei Ji, Zucheng Huang, Junhao Liu, Jianting Chen, Qingan Zhu

**Affiliations:** 1 Department of Spinal Surgery, Nanfang Hospital, Southern Medical University, Guangzhou, China

## Abstract

Translaminar screw fixation (TSF) of the axis is considered as an efficient, safe and simple surgical procedure, however the study of the potential risk of TSF to the transverse foramen in lower cervical spine is lacked. Head-neck CT images of 60 patients were included in this study. Maximum screw length, laminar thickness, the screw angle and the laminar height were measured. The feasibility of 3.5-mm diameter screw fixation and the potential risk of transverse foramen injury was analyzed. The TSF was safe at C3 and C4, but risky to the transverse foraman at a rate of 8.7% at C5 (0% on the left side and 20% on the right side), 33.3% at C6 (24.4% on the left side and 42.9% on the right side). C7 had the highest 77.8% rate (65.5% on the left side and 89.8% on the right side). The safe screw length was 27.7 mm at C3, 27.4 mm at C4, 28.0 mm at C5, 25.6 mm at C6 and 25.5 mm at C7, respectively. The present study showed that translaminar screw could place the transverse foramen of C5–C7 at risk. Preoperative CT scanning was necessary for safe screw placement.

Translaminar screw of C2, firstly described by Wright^1^, is considered to reduce the risk to the vertebral artery (VA) and nerve root, and make relevant structures visualized during operation^2^,^3^. Several studies showed C2 translaminar screw was similar to C2 pedicle screw and C2 pars screw in biomechanical performance^4^,^5^,^6^. Recent clinical studies reported a lower probability of ventral cortical breaches or instrumentation failures with C2 translaminar screw fixation, and a higher fusion rate without neural or vascular complications^1^,^2^,^3^,^7^,^8^,^9^,^10^,^11^,^12^. However, a computerized tomography angiogram–based morphometric analysis conducted by Riesenburger RI *et al*.^13^ showed that C2 translaminar screws could jeopardize the vertebral arteries in the foramen transversarium or the C1–2 interval.

Recent years, translaminar screw technique has been increasingly used in the lower cervical spine and upper thoracic spine, and previous studies have demonstrated the feasibility of translaminar screw placement in the lower cervical laminae^10^,^13^,^14^,^15^,^16^,^17^,^18^,^19^,^20^,^21^,^22^,^23^,^24^,^25^,^26^,^27^. The second segment of vertebral artery courses through the transverse foramen (TF) from C6 to C1 (3.95% of cases enter to the TF at the level of C7)^28^,^29^. In theory, a translaminar screw may breach the TF depending on the anatomical variation and fixation level, and the possible concomitant VA injury may result in some serious applications, such as hemorrhage, neurologic sequelae, and even death^29^,^30^,^31^. However, to our knowledge, no literature is available on the risk of TF violation of lower cervical translaminar screws.

The objective of the present study was to evaluate the risk of translaminar screw fixation to the TF of cervical spine based on CT images, and determine a translaminar screw length for lower cervical spine.

## Methods

After the institution review board approved the study, we retrospectively analyzed patients who presented to the Department of Spinal Surgery at Nanfang Hospital between July 2014 and January 2015. The adult patients with cervical spondylotic myelopathy or cervical spondylosis radiculopathy, requiring thin layer CT scan of the cervical spine were included. Patients were excluded if they had any congenital deformities, history of spinal surgery or traumatic. We firstly enrolled 69 cases, and 9 cases did not meet inclusion criteria (1 case with atlas assimilation, 6 cases with posterior expansive open-door laminoplasty, 2 cases with traumatic fracture). A total of 60 adult patients (21 cases with cervical spondylotic myelopathy, 39 cases with cervical spondylosis radiculopathy) with mean age of 57.5 ± 15.1 years was available for analysis.

Images were obtained from CT scans (Philips Brilliance 16 CT; Philips Medical Systems, Eindhoven, The Netherlands) with slice thickness of 1.0 to 1.5 mm, collimation of 0.75 to 1.5 mm, pitch of 0.7 mm, 120 kV, 180 mA, 512 × 512 matrix, and reconstruction level of 1 mm. Images of the sagittal and axial planes of the CVJ region were obtained after multiplanar reconstruction on the workstation (MXV, Philips). Bone windows were used for analysis.

**Figure 1:**
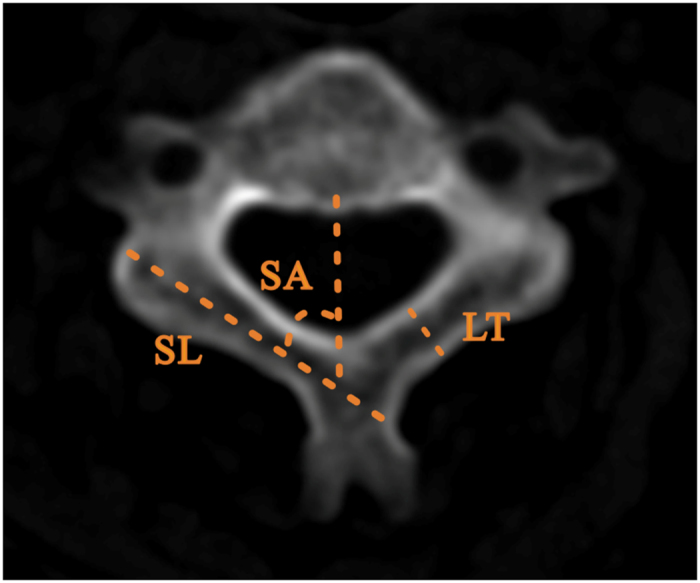
Measurement of the lanimae of lower cervical spine on CT image. In the axial plane, maximal screw length measurement (SL), the screw angle measurement (SA), and laminae thickness measurement (LT).

**Figure 2:**
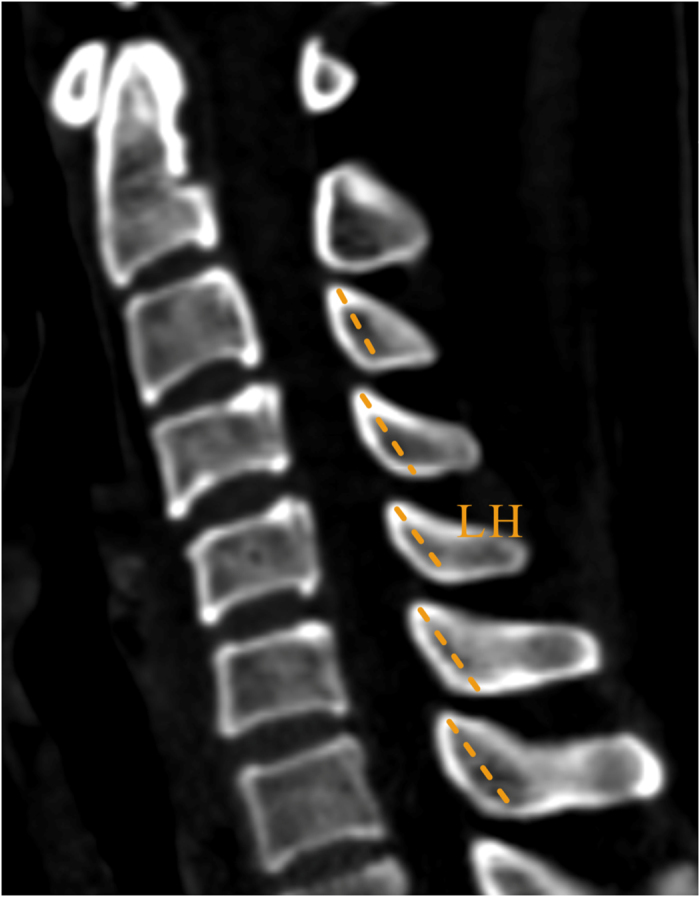
Measurement of the lanimae of lower cervical spine on CT image. In the sagittal plane, laminar height measurement (LH).

The translaminar screw entered the lamina from the junction of the lamina and spinous process, then traversed the lamina and entered the lateral mass. In the axial plane, maximal screw length (SL) was measured from the junction of the lamina and spinous process to the contralateral outer cortex of the lateral mass; The screw angle (SA) was measured between a line dropped from the midpoint of the vertebral body to the spinous process and the line of the screw trajectory; laminar thickness (LT) referred to the measurement of the narrowest portion of the lamina (Fig. 1). In the sagittal plane, laminar height (LH) was measured as the height of the junction of the lamina and the spinous process (Fig. 2)^13^,^24^,^25^. For unilateral placement, the thickness of lamina should be greater than 3.5-mm, and for placing the bilateral screws, we assumed a minimum required laminar height of 7 mm.

**Figure 3:**
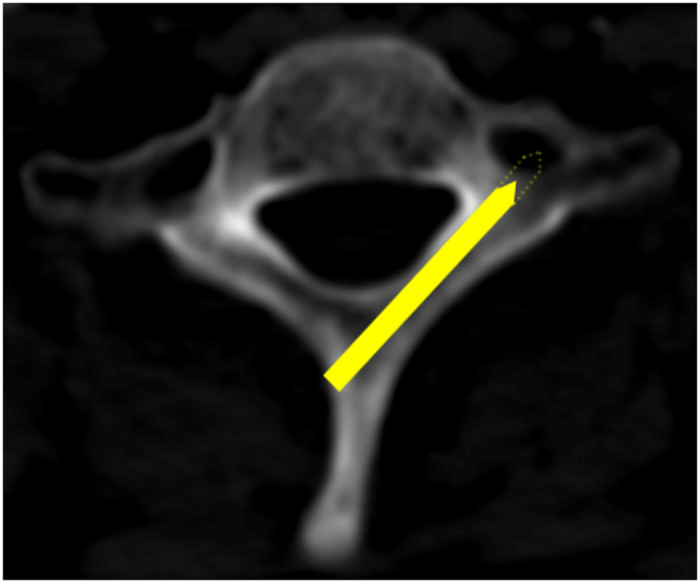
Axial views of the lamina that was deemed as “at risk”. A relative long screw in the anatomically defied screw trajectory could place the transverse foramen at risk (C7).

**Figure 4:**
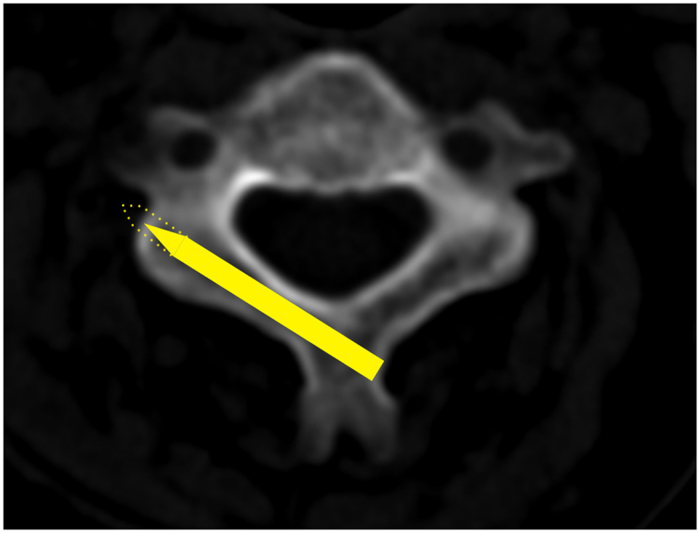
Axial views of the lamina that was deemed as “not at risk”. The anatomically defied screw trajectory could not place the transverse foramen at risk (C3).

For the lamina which was feasible for a 3.5-mm screw along the axial direction of the lamina, a dotted line extended from the screw was drawn. If the extension dotted line passes into the TF, the lamina was defined as “at risk” as the wall of TF may be broken with a longer screw^13^ (Fig. 3); if not, the lamina was regarded as “not at risk” (Fig. 4). At each vertebral level of the cervical spine, the minimum screw length of all the 60 cases was recommended as the safe length.

Two observers blinded to clinical information performed CT morphometric measurements. The intraobserver and interobserver reliability were calculated, for each parameter in 1 set of 15 patients who were randomly selected at 3-week intervals, using the intraclass correlation coefficient.

The statistical analysis was conducted by SPSS 20.0 software (IBM, USA). Values were represented as mean ± standard deviation. Single-factor analysis of variance and LSD multiple comparison were used to compare the measurement data among different vertebral levels. The student *t-*test was used to determine differences between different groups (female vs. male, and left side vs. right side). Pearson chi-square test and Fisher’s exact test were used to compare the virtual screw placement acceptance rates and potential risk rates between different groups. Spearman’s correlation coefficient was calculated to determine the correlation between the risk rates with SA. Differences were considered to be significant at a level of P < 0.05.

## Results

A total of 60 patients (35 men and 25 women) were available for this study. The mean age was 57.5 ± 15.1 years (range: 21–84 years).

### Anatomical analysis

There was no significant difference of SL between vertebra levels (Table 1). The mean SA decreased from C3 to C7. The mean LT decreased from C3 to C4 and increased from C4 to C7. The mean LH decreased from C3 to C5 and increased from C5 to C7. There was no significant difference in SL, SA and LT between the right and the left lamina at each vertebral level. And no significantly differences were found in SA and LH between genders at each vertebral level. At C6, however, men had significantly longer SL than women (P = 0.007). At C6 and C7, men had significantly larger LT than women (C6 P = 0.05 and C7 P < 0.001).

### Feasibility of lower cervical translaminar screw placement

Unilaterally, C7 showed acceptance rate above 96%, C3 and C6 showed acceptance rates more than 60%, C4 and C5 showed acceptance rates below 45% (Table 2). There was no significant difference (P > 0.05) between the right and the left lamina at each vertebral level. The bilateral acceptance rates of C3–C7 were 55%, 26.7%, 30%, 66.7% and 96.7%, respectively. The significant difference of bilateral acceptance rates was only found at C3 (P = 0.12) when compared between the genders.

### Risk rate to the transverse foramen

**Figure 5:**
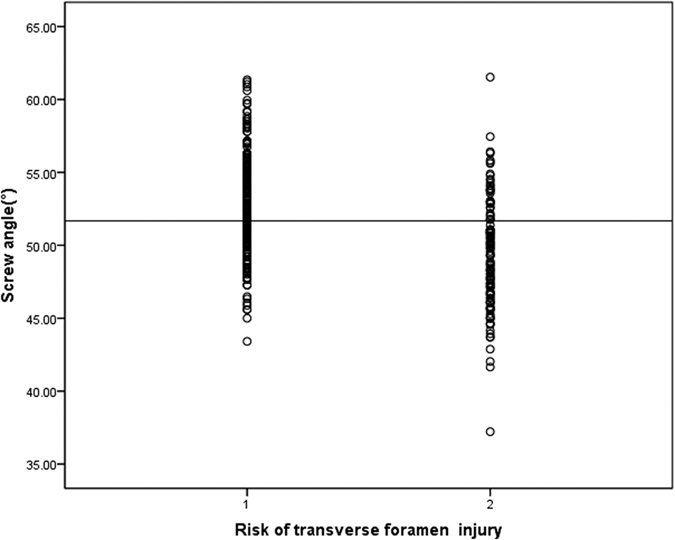
Distribution of screw angle stratified by whether the transverse foramen was at risk for injury(r = −0.425, P < 0.001). 1 not at risk, 2 at risk.

The potential risk for transverse foramen was not noted at C3 or C4 (Table 3), but the total risk rate of C5 was 8.7% (0% on the left side and 20% on the right side), C6 had a 33.3% risk rate (24.4% on the left side and 42.9% on the right side), and C7 had the highest 77.8% rate (65.5% on the left side and 89.8% on the right side). A significant difference between the left and right side was only found at C7 (P = 0.02). Genders did not have a significant influence of the potential risk rate in C5–C7. There was a significant correlation between risk rate and SA (r = −0.425, P < 0.001. Fig. 5).

### Recommended safe screw length

The minimum screw length at each vertebral level was recommended as the safe screw length. In our study, the recommend safe screw length from C3–C7 was 27.7 mm, 27.4 mm, 28.0 mm, 25.6 mm and 25.5 mm, respectively (Table 1).

The intraobserver reliability ranged from 0.91 to 0.95 for the initial examiner and from 0.90 to 0.96 for the secondary observer. The interobserver reliability ranged from 0.81 to 0.97.

## Discussion

Posterior cervical screw fixation was applied to stabilize the cervical spine in deformity, degenerative, trauma, tuberculous spondylitis and tumor reconstructive surgeries, and it can be accomplished through a variety of screw techniques including transarticular screws, lateral mass screws, pedicle screws, and, more recently, translaminar screws. Lateral mass screws are relatively safe and easy constructs to insert, but screw loosening or avulsion has still been reported as a failure mechanism for lateral mass screws because of the lower pullout strength. Pedicle screw fixation is the most biomechanically stable technique, but they may carry a high risk of neurovascular complications, and can be technically difficult^20^,^26^. Hooks fixation is often used in elderly or osteoporotic patients, it has less risky to vertebral artery than screw fixation at some levels. However, the use of hooks in the cervical spine has been restricted, especially at the stenotic levels, because hooks imposed additional stresses on the vertebrae, had lower maximum failure strength than screws, and presented additional risk of spinal cord compression leading to the clinical tetraplegia^32^,^33^. In contrast, translaminar screw fixation is purported to eliminate the risk of neurovascular structures as the screw can be placed under direct vision of the outer cortex of the lamina and all relevant structures, and able to yield high clinical efficacy based on the published series. With respect to biomechanics, translaminar screws may not perform as well *in vitro* as other techniques, but it may be a suitable salvage technique should pars or pedicle screw fixation fail^34^. The surgeon may choose the translaminar method as an alternative to traditional fusion constructs, because it is considered as a less technically demanding and safer technique.

However, the present study showed translaminar screws may jeopardize TF in the lower cervical spine depending on individuals and vertebral levels. The risk of translaminar screws was higher at C6 and C7, but free at C3 and C4. Moreover, the highest acceptance rate of the translamiar screws was in C7, while the lowest in C4.

Riesenburger RI *et al*.^13^ found potential risk for VA injury 55% at C2. And we analyzed the potential risk of translaminar screw fixation to the TF of lower cervical spine in the present study, no risk was noted at C3 and C4, the total risk rate of C7 was the highest (77.8%), C6 was lower (33.3%), and C5 was the lowest (8.7%). The different risk rates may depend on the anatomical structure of different vertebral levels. Firstly, the SA of C6 was significantly smaller than C3, C4 and C5, respectively, it was the same with C7. In another word, the laminae of C6 and C7 were closer to the vertebral body. Secondly, the previous literature had demonstrated the area of TF increased from C3 to C6 vertebra (20.92 ± 3.78 mm^2^ at C3, 22.48 ± 8.19 mm^2^ at C4, 26.40 ± 4.05 mm^2^ at C5, 28.92 ± 8.16 mm^2^ at C6)^35^. We also found a significant correlation between risk rate and SA (r = −0.425, P < 0.001).

Feasibility of lower cervical translaminar screw placement based on computerized tomographic measurements was studied by Alvin MD *et al*.^24^, and they reported C4 and C5 never accepted bilateral translaminar screw, C3 and C6 accepted bilateral screws at low placement rates (8–24%), C7 accepted bilateral placement at a high rate (96% men, 84% women). However, a cadaveric study of 37 spines conducted by Yusof MI *et al*.^25^ showed that approximately 30, 17, 18, 49 and 100% of patients may receive translaminar screw fixation at C3–C7, respectively. These were similar with our current results, the present study showed the bilateral acceptance rate of C7 (96.7%) was the highest, C3 (55%), C5 (30%) and C6 (66.7%) were lower, and C4 (26.7%) was the lowest, which proved that C7 had the best acceptance rate.

Consequently, our results indicate that the spine surgeon should review CT scan images preoperatively to evaluate the safety and feasibility, and to determine the optimal screw length, angle, ideal entry points and trajectories, especially when a longer screw will be placed at C5–C7. To avoid the injury to the TF, adequate screw length and a larger screw angle were recommended for C5–C7 translaminar screw placement. We recommended that the safe length of the translaminar screw from C3–C7 were 27.7 mm, 27.4 mm, 28.0 mm, 25.6 mm and 25.5 mm, respectively. At C3 and C4, the screw with appropriate length could avoid damage to the outer cortex of the lateral mass, and at C5–C7, to the outer cortex of the lateral mass and the wall of TF. Ventral spinal canal violations have been considered as a major drawback of translaminar screw^3^, and a biomechanics study conducted by Claybrooks R. *et al*.^36^ showed that TSF had less rigidity in lateral bending and axial rotation compared with pedicle screw fixation. To avoid these defects, Wright’s technique was modified to have an “exit” window on the dorsal aspect of the lamina by Jea A. *et al*.^37^, the tip of the screw exiting the posterior cortex from this window could be observed clearly. Meanwhile, it assured bicortical fixation and possibly increased the biomechanical stiffness of Wright’s technique. And the modified technique could also eliminate the risk for TF, because of the directly visualizing the exact position of the screw tip in the “exit” window. However, the screw tip out of the “exit” window may damage the adjacent soft tissue. In our study, a larger screw insertion angle which meant putting the screw tip close to the dorsal cortex of the lamina was recommended to avoid the damage to transverse foramen, vertebral artery and spinal canal. And it should be noted that the angle was limited by the lamina boney structure defined translaminar screw trajectory. To avoid the damage to the soft tissue, the screw with appropriate length was necessary, and the screw tip should be purchased in the lamina. Furthermore, three-dimensional (3D) fluoroscopy-based navigation was recommended to enhance the accuracy and safety of the technique during operation^38^.

There were some limitations of this study. Our study population was comprised of 60 patients all come from China and may not have been sufficiently large to be generalized to other ethnic groups. We only used CT images to evaluate the potential risk to TF of lower cervical translaminar screw, and the measurements may not predict clinical outcomes directly.

## Conclusion

Lower cervical translaminar screw placement was a reliable alternative to other earlier techniques, but at C5–C7, it could still place the transverse foramen or even the vertebral artery at risk. We suggested that preoperative CT scanning was obligatory for all vertebral levels. Adequate screw length and a larger screw angle were recommended especially during C5–C7 translaminar screw placement.

## Additional Information

**How to cite this article:** Kong, G. *et al*. The risk of translaminar screw fixation to the transverse foramen of the lower cervical spine: a computed tomography study. *Sci. Rep.* **7**, 46611; doi: 10.1038/srep46611 (2017).

**Publisher's note:** Springer Nature remains neutral with regard to jurisdictional claims in published maps and institutional affiliations.

## Figures and Tables

**Table 1:** Anatomic analysis of C3–C7 vertebral laminae.

Vertebral level	Screw length (mm)	Screw angle (°)	Lamina thickness (mm)	Laminar height (mm)
C3	31.8 ± 2.3(27.7–38.2)	53.3 ± 3.5(43.4–61.4)^de^	3.9 ± 1(1.9–6.8)^bcde^	12 ± 1.6(8.8–14.7)^de^
C4	32.6 ± 2.3(27.4–37.6)	53.3 ± 2.9(47.7–59.7)^de^	3.3 ± 0.8(1.6–5.3)^ade^	11.7 ± 1.7(8.5–14.8)^de^
C5	32.9 ± 2.2(28–38.8)	52.7 ± 3.2(45.1–59.8)^de^	3.4 ± 0.8(2–6)^ade^	11.1 ± 1.3(7.8–12.5)^de^
C6	31.9 ± 2.6(25.6–37.9)	51 ± 4.1(37.2–61)^abc^	4.2 ± 1(2.4–7.5)aBCE	12.8 ± 1.4(9.5–15.7)^abce^
C7	32.4 ± 2.7(25.5–39.1)	50.1 ± 3.5(41.7–61.5)^abc^	6.2 ± 1.5(2.5–11)^abcd^	14.8 ± 1.7(11.2–19.1)^abcd^

Data are expressed as mean ± standard deviation (range).

^a^Significant difference between analysis vertebral level and C3(p < 0.05).

^b^Significant difference between analysis vertebral level and C4(p < 0.05).

^c^Significant difference between analysis vertebral level and C5(p < 0.05).

^d^Significant difference between analysis vertebral level and C6(p < 0.05).

^e^Significant difference between analysis vertebral level and C7(p < 0.05).

Capital letters means the difference is significant at the 0.01 level.

**Table 2:** Acceptance rates of translaminar screw at C3–C7.

Vertebral level	C3	C4	C5	C6	C7
Unilateral L	66.7% (40/60)	41.7% (25/60)	43.3% (26/60)	75% (45/60)	96.7% (58/60)
Unilateral R	63.3% (38/60)	31.7% (19/60)	33.3% (20/60)	70% (42/60)	98.3% (59/60)
Bilateral	55%* (33/60)	26.7% (16/60)	30% (18/60)	66.7% (40/60)	96.7% (58/60)

L means the left side of the lamina.

R means the right side of the lamina.

*Means significant difference between the genders.

**Table 3:** The risk rates to the transverse foramen.

Vertebral level	C3	C4	C5	C6	C7
Unilateral L	0% (0/40)	0% (0/25)	0% (0/26)	24.4% (11/45)	65.5%*(38/58)
Unilateral R	0% (0/38)	0% (0/19)	20% (4/20)	42.9% (18/42)	89.8% (53/59)
Total	0% (0/78)	0% (0/44)	8.7% (4/46)	33.3% (29/87)	77.8% (91/117)

L means the left side of the lamina.

R means the right side of the lamina.

*Means significant difference between the left and right side.
